# TheraCal LC versus Mineral Trioxide Aggregate for full pulpotomy in cariously exposed mature permanent molars with symptomatic irreversible pulpitis: a randomized clinical trial

**DOI:** 10.2340/biid.v13.45702

**Published:** 2026-04-21

**Authors:** Vignesh Ravi, Ritu Namdev, Ruchi Singhal, Madhur Sharma, Charu Dayma, Asha Rani

**Affiliations:** a Department of Pediatric and Preventive Dentistry, Post Graduate Institute of Dental Sciences, Pandit Bhagwat Dayal Sharma University of Health Sciences, Rohtak, Haryana, India; b Department of Oral and Maxillofacial Pathology, Post Graduate Institute of Dental Sciences, Pandit Bhagwat Dayal Sharma University of Health Sciences, Rohtak, Haryana, India

**Keywords:** MTA, TheraCal LC, pulpotomy, irreversible pulpitis, mature permanent molars

## Abstract

**Introduction:**

Preserving pulp vitality is crucial for long-term tooth survival. Pulpotomy, which involves the removal of infected pulp while retaining healthy pulp, helps maintain tooth vitality and function. Although TheraCal LC has shown success as a pulp-capping agent, its use in pulpotomy remains unexplored. This study compared the clinical and radiographic success of TheraCal LC and mineral trioxide aggregate (MTA) in full pulpotomy of mature permanent molars in young patients with symptomatic irreversible pulpitis (SIP).

**Materials and methods:**

In this double-blinded, parallel, non-inferiority randomized controlled trial, 72 healthy children (aged 9–14, mean age = 11.72 ± 1.35 years) with cariously exposed mature permanent molars diagnosed with SIP were randomly assigned to receive either TheraCal LC or MTA (*n* = 36 each). Follow-ups were conducted up to 12 months. Data were analyzed using Mann-Whitney U, Chi-square, and Kaplan-Meier tests (α = 0.05).

**Results:**

At 12 months, MTA showed significantly higher success (ITT – Intention to Treat: 94.4%, PP – Per Protocol: 100%) compared to TheraCal LC (ITT: 75%, PP: 79.4%) (*p* < 0.05). Seven failures occurred in the TheraCal LC group; none in the MTA group. Two patients in each group were lost to follow-up. Pain scores were significantly lower in the MTA group on days 1, 3, and 5 (p < 0.05).

**Conclusions:**

MTA demonstrated superior outcomes over TheraCal LC for full pulpotomy in young, mature permanent molars with SIP.

**Statement of clinical relevance:**

The use of MTA in pulpotomy procedures for mature permanent molars with SIP results in enhanced clinical success rates when compared to TheraCal LC, offering valuable insight for evidence-based treatment planning.

**KEY POINTS**

1. Vital pulp therapy is successful in the treatment of symptomatic irreversible pulpitis condition of cariously exposed mature permanent teeth.
2. According to this study, MTA is superior to TheraCal LC as a pulpotomy agent.

## Introduction

Preserving the integrity of the dental pulp is the basic objective of all restorative therapies [1]. The innate defenses and abundant blood flow of the young pulp confer enhanced resistance to bacterial infection [2]. During the initial stages of pulpal inflammation, the inflammatory changes are typically limited to the coronal pulp, whereas the remaining pulp tissue remains ­relatively healthy [3].

The traditional treatment modality for permanent mature teeth with symptomatic irreversible pulpitis (SIP) is nonsurgical root canal treatment (NSRCT). When NSRCT is performed on teeth with SIP, 92% to 98% of the teeth have a high success and survival rate [4–6]. However, NSRCT has several disadvantages, including being time-consuming, expensive, requiring multiple visits, and making the teeth susceptible to fractures [7]. For the last few decades, an alternative to NSRCT for teeth with pulp inflammation has been vital pulp therapy (VPT), a minimally invasive technique [1, 5].

VPT is indicated in patients with traumatic, carious, or mechanical pulp exposure who do not exhibit any periapical rarefaction with an intact lamina dura. VPT aims to maintain tooth vitality and functionality, along with rendering the tooth asymptomatic [8]. VPT is completed in a shorter appointment in a single visit [9]. Several studies have shown that cariously exposed pulps of mature teeth are capable of healing, and VPT need not be restricted to young or asymptomatic teeth. Furthermore, the presence of spontaneous or severe preoperative pain and extremely deep carious lesions is not unconditionally related to an irreversible pattern of pulpal pathology [10–12]. The rationale behind full pulpotomy procedures is based on the ability of the remaining radicular pulp to recover after placement of a suitable medicament [13]. The ability to control bleeding after amputation of the infected pulp tissue has been suggested as a surrogate marker for the degree of inflammation and the healing potential of the remaining pulp tissue [14].

The type of material used for pulpotomy is another factor that determines the success of pulpotomy. Hydraulic calcium silicate cements (HCSCs) are advocated as the ‘gold standard’ material for pulpotomy procedures [15]. Mineral trioxide aggregate (MTA) is one of the most commonly used and researched materials with successful clinical outcomes. However, due to certain inherent drawbacks, such as extended setting time, handling, and tooth discoloration, there is a need for the development of newer materials [16].

TheraCal LC creates a new category of light-cured resin-modified calcium silicate in a hydrophilic monomer. This hydrophilic matrix provides significant calcium release, creating an alkaline, sustained environment that promotes dentin–pulp complex healing and regeneration [17]. Histological evaluation revealed that 80% of the teeth exhibited normal pulp organization, accompanied by mild to moderate chronic inflammatory cell infiltration in those teeth treated with TheraCal LC. Furthermore, 86.66% of cases demonstrated complete dentin bridge formation, indicating effective hard tissue barrier development [18]. Although disruption of the odontoblastic layer was observed in some areas, undifferentiated mesenchymal cells were present along the root canal walls, contributing to osteodentin formation [3, 17]. These findings support the bio-inductive potential of TheraCal LC, while also highlighting localized variations in pulpal healing response. Hence, it is advocated for use as a pulp-capping agent and as a protective liner.

Therefore, to explore a material that could overcome the drawbacks associated with MTA, this study was designed to evaluate TheraCal LC as a material for full pulpotomy in mature permanent molars in 9–14-year-old patients with deep carious lesion diagnosed with SIP and compare it to the success rate achieved by MTA, clinically and radiographically up to 12 months.

## Materials and methods

This randomized clinical trial has been written according to Preferred Reporting Items for Randomised Trials in Endodontics (PRIRATE) 2020 guidelines [19, 20].

### Trial design

A double-blinded, parallel, non-inferiority, randomized controlled trial.

### A priori protocol

The present study was approved by the Biomedical and Health Research Ethics Committee via ethical no. PGIDS/BHRC/22/44 dated 25/06/2022, and the study was registered at the clinical trials website (http://www.clinicaltrials.gov) with the number (NCT05733468). All procedures were performed following good clinical practice guidelines and the Declaration of Helsinki.

### Participants

Subjects in the age group 9–14 years were selected from the pool of patients visiting the outpatient department as per the inclusion and exclusion criteria (Table 1) for full pulpotomy. Informed consent was obtained from the parents or legal guardians of patients, and an assent form from the patient.

**Table 1:** Inclusion and exclusion criteria for the study groups.

Inclusion criteria	Exclusion criteria
Patient should be between 9 and 14 years of age. Intermittent or spontaneous, sharp or dull, localized, diffuse, or referred pain. Permanent first molar tooth with deep caries and subsequent pulp bleeding evident upon excavation of caries. Rapid exposure to dramatic temperature changes elicited heightened and prolonged episodes of pain even after removal of the thermal stimulus. Vital bleeding pulp tissue should be present in all canals after complete pulpotomy. Diagnosis should be consistent with symptomatic irreversible pulpitis. The tooth is restorable and free from advanced periodontal disease. Normal soft tissues around the tooth with no swelling or sinus tract. Hemostasis should be achieved after complete pulpotomy Non-contributory medical history.	Teeth with immature roots. Non-restorable. Pathological mobility. Pus discharge through an associated sinus tract. Swelling of associated tissues. Radiographic internal or external resorption or any periapical rarefaction including PDL widening. Necrotic pulp upon exposure. Bleeding beyond 25 min. Children with severe systemic illness (mental retardation/severe psychotic disorders), prior history of allergy and any medical condition not permitting the intervention. Parents and patients unwilling to participate in the study.

PDL: Periodontal Ligament.

### Sample size calculation

The sample size was determined using the expected proportion of event/outcome in each group, values of which were estimated from literature NA Taha et al. [21] (π_S_-0.96) and Peskersoy et al. [22] (π_C_ -0.73) and the formula for calculating the sample size was obtained from Bouman AC et al. [23]. With a 2% non-inferiority limit, 80% power, and 5% significance level, the required sample size was 66. A minimum of 10% of the required sample size was added to compensate for follow-up loss, resulting in 72 subjects in total, with 36 per group.

### Randomization/allocation concealment/blinding

**Figure 1:**
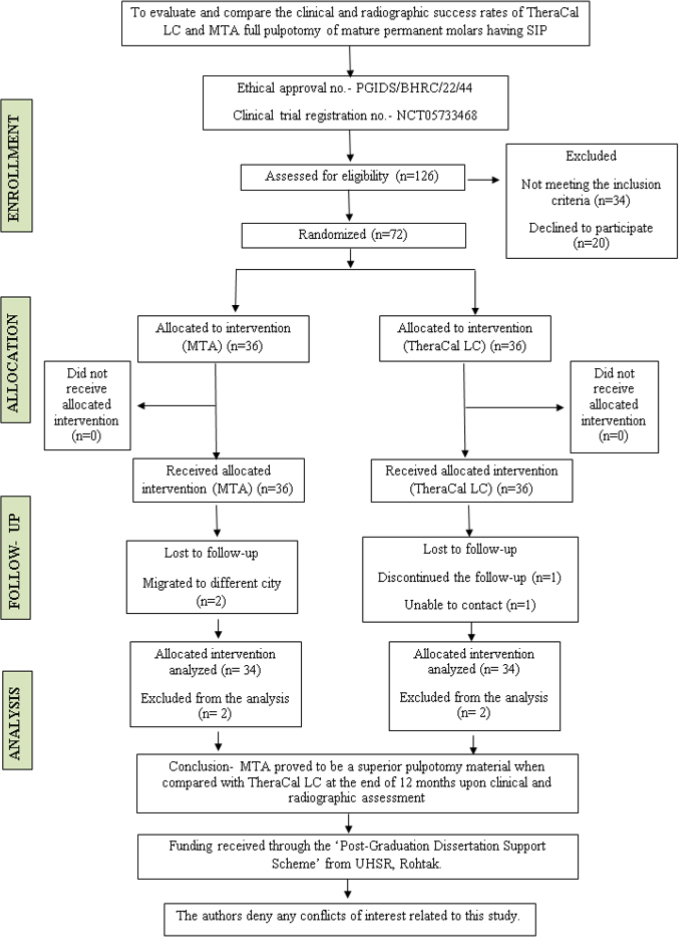
PRIRATE 2020 flow chart of participants throughout the trial.

Randomization was carried out for each patient. Block randomization was employed with a block size of 4 with a 1:1 allocation ratio. Using sealed envelopes, the sequence concealment was carried out. Unaware of the study group assignment, an independent person who is not part of the study recruited the participants. Only one tooth was included per patient. Both the patient and the assessor who evaluated the results were blind to the research groups. This study is double-blinded, as it was impossible to blind the operators because each pulpotomy agent had a different technique of manipulation; however, the patient and assessor were blinded due to the similar radiodensity property of the materials used (Figure 1).

### Clinical intervention

A total of 72 teeth in 72 patients were treated between July 2022 and December 2022 at the outpatient department. After obtaining informed consent, a brief history of the chief complaint and a clinical examination, including visual inspection of caries status, restorability of the tooth, percussion, palpation, and sensibility via cold testing (Endo Ice F, Coltene Whaledent, USA) were performed.

According to the AAE 2021/2022 guidelines [24], a diagnosis of SIP is was established based on the presence of lingering pain, spontaneous pain, and referred pain on stimulation, and was followed by the acquisition of preoperative periapical radiographs.

All the endodontic procedures were performed by a single well-trained postgraduate student. The teeth were anesthetized using lidocaine 2% with epinephrine 1:100000 (ICPA Health Products Ltd, Ankleshwar, India) and isolated with a rubber dam. Access to the pulp chamber was obtained after the removal of caries and exposure of the vital pulp, using a high-speed bur with a water spray. Subsequently, the coronal pulp tissue was removed down to the canal orifices. A sterile cotton pellet dampened in sterile saline was placed against the pulp stumps at the orifices of the root canals. The bleeding from the pulp stump was evaluated every 5 min and monitored to determine whether hemostasis was achieved within 25 min of pressure application, as outlined in the study by Qudeimat et al. [2]. After hemostasis, the test material was placed as per the study groups. If the bleeding duration exceeded 25 min, the case was excluded from the study, and NSRCT was performed.

**Figure 2:**
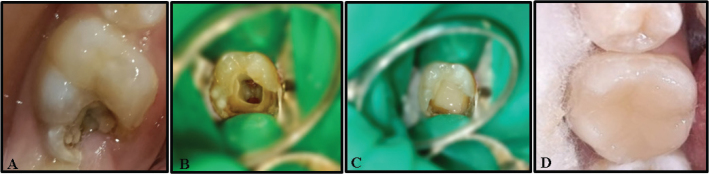
Steps for the clinical procedure. A-Cariously involved permanent molar. B-Bleeding control after full pulpotomy. C-Placement of pulpotomy material. D-Final restoration.

After hemorrhage control, 2–3 mm of MTA (MTA Angelus® White, Angelus Lodrina, Parana Brazil) was covered by a moist cotton pellet, and 1–1.5 mm of TheraCal LC (BISCO Dental Products, Schaumburg, IL, USA) was placed and light-cured on the radicular pulp stumps of the respective groups. Subsequently, the area was sealed with glass ionomer cement (Vitrebond, 3M Dental Products Division, St. Paul, MN, USA), and a direct composite restoration (Valux Plus, 3M ESPE, St. Paul, MN, USA) using Adper Single Bond 2 (3M ESPE, St. Paul, MN, USA) was placed in the same visit, once the pulpotomy materials were set (Figure 2). The participants were evaluated for postoperative signs and symptoms in the follow-up period.

**Figure 3:**
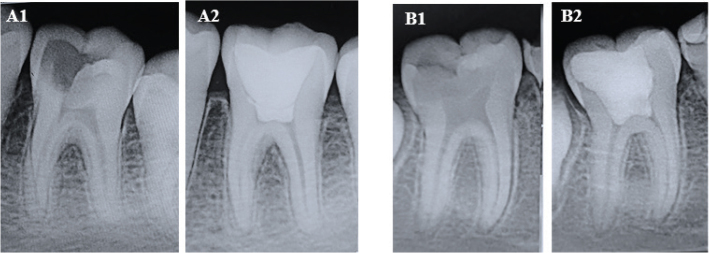
Pre-operative and post-operative radiographs at 12 months for MTA group. Case 1 (A1, A2); Case 2 (B1, B2).

**Figure 4:**
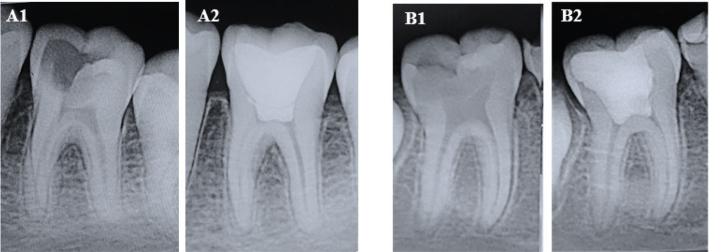
Pre-operative and post-operative radiographs at 12 months for Theracal LC group. Case 1 (A1, A2); Case 2 (B1, B2).

All procedures were performed under aseptic conditions, and periapical radiographs were taken. Standardization of the radiographs was done according to American Dental Association guidelines to have reproducibility during the follow-up period for evaluating the periapical changes post-treatment (Figures 3 and 4).

### Outcome measures

Preoperative evaluation of pain was done using a 10-point visual analog scale (VAS) by the operator. After the pulpotomy was done, the patient’s parent/guardian was given the scale and explained visually, verbally, and numerically to facilitate its use on evaluating the postoperative pain using VAS, and the assessment of the same was done telephonically through the patient’s parent/guardian at an interval of the first 24 h, 3^rd^ day, 5^th^ day and 7^th^ day. Bleeding time was categorized into 0–5 min group, 6–10 min group, etc., respectively, in both groups till 25 min, and was compared to the overall success rate. All the cases were evaluated clinically at 1 week postoperative, 1, 3, 6, and 12 months and were classified as failure if any signs and symptoms were present. Similarly, radiographic evaluation was done after 6 and 12 months (Table 2). An intraoral periapical radiograph was obtained using a digital imaging system (Carestream RVG 5200; Carestream Health Inc) by a digital sensor and sensor holding device with standard exposure parameters. During the follow-up visits, the baseline and follow-up radiographs of the teeth were evaluated by the conventional pairwise visual reading by two calibrated examiners, blind to the pre- and postoperative status of the teeth. In cases with clinical or radiographic failure at any point during the study period, endodontic treatment was carried out with the patient’s consent.

**Table 2:** Outcome assessment criteria for success and failure.

Clinical assessment	Radiographic assessment
Pain Tenderness on percussion Swelling/abscess Sinus tract/fistula Pathological mobility	Furcation radiolucency PDL widening Pathological root resorption (internal/external) Periapical radiolucency

PDL: Periodontal Ligament

### Statistical analysis

The study data were compiled and saved in a Microsoft Excel® spreadsheet. Statistical analysis was performed using the SPSS 25.0 software (SPSS Science, Chicago, IL, USA). Kolmogorov-Smirnov test of normality showed non-normal distribution of data, and thus non-parametric tests were used for analyses of data. Descriptive statistics were used for categorical data. Mann-Whitney U and post hoc Wilcoxon signed-rank tests were used to compare the pain scale over 0 to 7^th^ day between the two groups. An interrater reliability test (Cohen’s kappa) was performed to assess the interpreter’s agreement on radiographic assessment. Chi-square test was used to compare the success rates between different groups. The level of statistical significance was established at *p*-value < 0.05. Statistical analysis for success rates was done using intention-to-treat (ITT) and per-protocol (PP) analysis. The PP analysis comprised patients who completed the 12-month follow-up, while the ITT analysis comprised all patients who were initially randomized to the treatment groups. Kaplan-Meier analysis was done along with the log-rank test to assess the difference in survival between the two groups.

## Results

Patients were recruited beginning on 01/07/2022, and the recruitment endpoint was achieved on 24/12/2022. The PRIRATE 2020 flow diagram (Figure 1) shows that 126 cases were initially recruited and 54 cases were excluded because they did not meet the inclusion criteria. Seventy-two cases were then randomly allocated to the MTA (36 cases) and TheraCal LC (36 cases) groups. Two patients from each group did not report for any ­follow-up, resulting in an overall recall rate of 94.4% (68/72). At a mean follow-up period of 12 months, a total of 68 cases with 34 in the MTA group and 34 in the TheraCal LC group were analyzed. Baseline characteristics of the study participants are shown in Table 3. A total of 41 males and 31 females with an age range of 9–14 years and a mean age of 11.8 ± 1.37 years in the MTA group and 11.6 ± 1.29 years in the TheraCal LC group were included in the study. The gender differences in both groups were non-significant (*p* > 0.05). Neither the type of intervention nor the clinical and radiographic success was significantly impacted by confounding variables, including age, gender, the tooth or arch treated, or the molar treated (*P* > 0.05).

**Table 3:** Baseline characteristics of the study participants.

Group	No. of cases (*n*)	Mean age ± SD (years)	Gender distribution	
			Males *n* (%)	Females *n* (%)
MTA	36	11.8 ± 1.37	19 (52.7%)	17 (47.3%)
TheraCal LC	36	11.6 ± 1.29	22 (61.2%)	14 (38.8%)

MTA: mineral trioxide aggregate.

The mean preoperative pain scores measured using the VAS scale were similar between the MTA (6.61 ± 1.47) and TheraCal LC (6.67 ± 1.37) groups, and the difference was non-significant (*p* > 0.05). However, pain reduction showed significant differences in both groups over the subsequent 1^st^, 3^rd^, and 5^th^ days (*p* < 0.05). The mean pain score displayed a greater reduction in the MTA group, with the score becoming zero by the 5^th^ day (*p* < 0.05). The mean pain score in the TheraCal LC group reduced to zero by the 7^th^ day (*p* < 0.05) (Table 4).

**Table 4:** Intergroup comparison of interappointment pain at baseline and over a 1-week follow-up.

Time period	Mean ± SD		*P*
	MTA	TheraCal LC	
Preoperative	6.61 ± 1.47	6.67 ± 1.37	0.859
1^st^ day	0.94 ± 1.45	2.08 ± 1.53	0.001*
3^rd^ day	0.28 ± 0.51	0.69 ± 0.82	0.018*
5^th^ day	0.00	0.28 ± 0.45	0.001*
7^th^ day	0.00	0.00	1.00

SD: standard deviation; MTA: mineral trioxide aggregate.

* Indicates significant difference.

**Figure 5:**
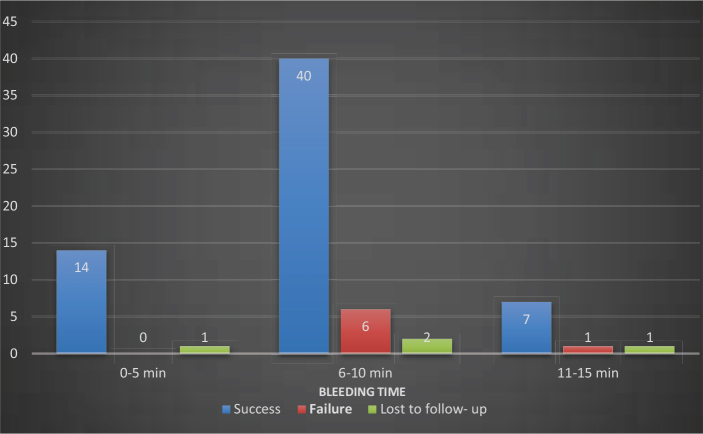
Number of success and failure cases when compared with bleeding time.

**Figure 6:**
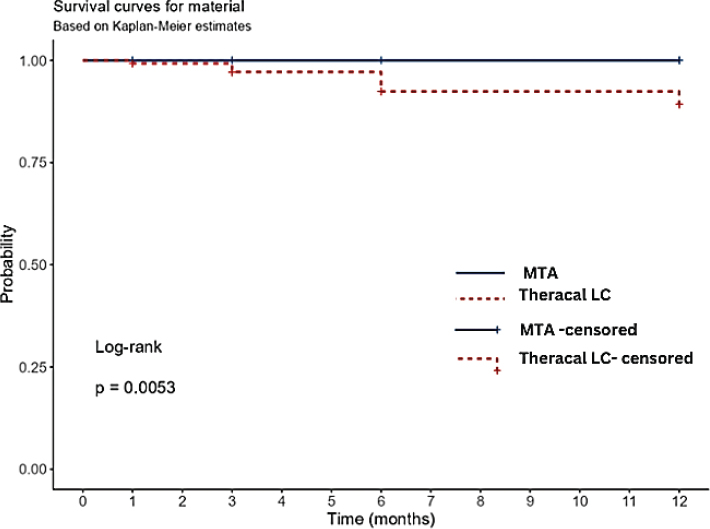
Kaplan-Meier curve depicting the cumulative survival probabilities of Theracal LC and MTA groups at 12-months follow-up.

Two cases in each group were lost to follow-up. Both of the lost cases of the MTA group migrated to a different state; one patient in the TheraCal LC group discontinued the follow-up, and one was unable to be contacted. Also, seven cases in the TheraCal LC group failed, where five patients reported with persistent pain and tenderness, one patient reported with swelling and sinus tract formation, while one patient had periapical radiolucency at different intervals of follow-up. No cases of failure were reported in the MTA group. None of the pulpotomy agent used in this study were found to have any adverse effects. According to ITT analysis, the 12^th^-month success rate was 94.4% in the MTA group, while 75% in the TheraCal LC group. In PP analysis, the 12^th^-month success rate for the MTA group was 100% and for the TheraCal LC group, it was 79.4% (Table 5) with a *p*-value < 0.05. Duration of bleeding time at the time of pulpotomy procedure was noted in both the groups, and the maximum number of individuals had a bleeding time of 6–10 min (Figure 5), and no significant difference between bleeding time and success rate was found (*p* > 0.05). Kaplan-Meier analysis with log rank test revealed that success rates of MTA and TheraCal LC groups at 12-month follow-up had a significant difference with a *p*-value of 0.0053 (Figure 6). An absolute risk difference (95% confidence interval) with ITT analysis was found to be 19.4% (3.4%, 35.4%) and with PP analysis was 20.6% (7.4%, 33.8%). The results of Cohen’s Kappa statistics showed good interobserver agreement of 0.90.

**Table 5:** Treatment outcome according to intention-to-treat (ITT) and per-protocol (PP) analysis.

Treatment group	Total teeth	Teeth available for follow-up at 12^th^ month	Success	Failure	ITT analysis			PP analysis		
					Success rate	Absolute risk difference (95% confidence interval)	*P*	Success rate	Absolute risk difference (95% confidence interval)	*P*
MTA group	36	34	34	0	94.4% (34/36)	19.4% (3.4% – 35.4%)	0.005*	100% (34/34)	20.6% (7.4% -33.8%)	0.005*
TheraCal LC group	36	34	27	7	75% (27/36)			79.4% (27/34)		

MTA: mineral trioxide aggregate.

* Indicates significant difference.

## Discussion

The dental pulp exhibits a remarkable capacity for healing and regeneration following the removal of infected and bacterially contaminated tissue [25]. In light of advancements in pulpal biology and the development of bioceramic materials, the concept of ‘ VPT’ has been reexamined. Clinical evidence shows that pulpotomy procedures in permanent teeth demonstrate success rates ranging from 82% to 100% [26].

The meta-analysis by Ather et al. revealed that pulpotomy has an 83% cumulative success rate when used on teeth with a closed apex, while teeth with an open apex showed a markedly better result. This is explained by the pulp’s higher vascularity and cellularity in teeth that are still developing [27]. Wang et al. demonstrated the presence of stem/progenitor cells in teeth with irreversibly inflamed pulp, suggesting that such teeth may retain a certain degree of regenerative and reparative potential [28]. Factors that determine the success of pulpotomy include the reparability of vital pulp and the biocompatibility of the pulp-capping agent used. Various studies support the use of MTA as a pulpotomy agent because of its excellent sealing ability, biocompatibility, and dentin bridge formation, in addition to inducing proliferation of the pulpal cells; however, it has a few limitations [8, 29].

The difference between the overall success rates of MTA and TheraCal LC was found to be significant. When the ITT analysis was done, the MTA group had an overall success rate of 94.4%, and TheraCal LC had an overall success rate of 75%. However, PP analysis revealed the overall success rate of MTA as 100% and TheraCal LC as 79.4%. Hence, based on the findings of the present study, MTA can be considered a better material than TheraCal LC in pulpotomy of permanent molars with irreversible pulpitis. Barngkgei et al. and Alqaderi et al. in their studies found similar results with the success rates of MTA as 100% and 90%, respectively [8, 9]. Additional studies by Qudeimat et al., Sharma et al., and Taha et al. contributed to evidence in favor of MTA while doing full pulpotomy [2, 21, 30].

TheraCal also showed less favorable results in the study by Lee et al. [31] This was attributed to increased cavity depth in pulpotomy treatment and limited access to the curing light. Thus, reduced polymerization degree might increase the level of uncured resin monomers, which eventually lowers the cement biocompatibility [32]. In a study by Bakhtiar et al., with a cavity depth of 2 millimeters, TheraCal showed poorer pulpotomy outcomes compared to MTA [33]. Other possible explanations for the limited success of TheraCal could be its external chemical nano-shell surface, which could mask the calcium ion release from these materials [34]. A study conducted to compare TheraCal LC, MTA-Angelus, and Biodentine found that TheraCal exhibited mild to moderate inflammation after 1 month and moderate to severe inflammation after 3 months. It was concluded that TheraCal LC is less biocompatible, and this might be due to the presence of resin in its composition [35]. Contrary to our findings, Zeater et al. [18] found similar effectiveness of both MTA and TheraCal LC in dentin bridge formation and subsequent success of pulp-capping procedures, while Shalini et al. [3] reported better effectiveness of TheraCal LC when compared to Biodentine in direct pulp capping. Hence, more studies are needed to explore the efficacy of this material as a pulpotomy agent.

Pain is the principal complaint for which an individual usually seeks endodontic treatment. Research carried out by Asgary et al. reported more and quicker pain relief with pulpotomy when compared to RCT [36]. In the present study, a significant reduction was found in the MTA group till the 5^th^ day, after which no patient reported pain. On the other hand, the TheraCal LC group displayed a significant reduction till the 7^th^ day. The mean pain score was seen to reduce more in the MTA group than the TheraCal LC group, thus proving MTA to be a better material when pain relief is considered. The authors believe that this might be due to the incompletely polymerized resin in TheraCal LC, which can act as an irritant and thus leads to prolonged pain when compared to MTA. Similar results were found by Beauquis et al. [37], who analyzed the evolution of pain at 24 h and 7 days follow-up and found a moderate drop in pain intensity after 24 h and a substantial reduction to minimal levels in 7 days. Hence, pulpotomy using tricalcium silicate–based cements is advocated as an effective treatment for irreversible pulpitis, providing short-term pain relief comparable to the gold standard.

The present study included subjects with an age range of 9–14 years, and the gender difference between the two groups was non-significant. In a randomized clinical trial by Taha et al. and Kang et al., it was concluded that gender was not a prognostic factor and did not have a significant effect [1, 38]. Contrary to these findings, Mass and Zilberman reported significant differences between males and females in hard tissue bridge formation and pulp horn obliterations during radiographic examination after partial pulpotomy [39]. Duncan et al. recommended that a separate analysis of the success rate of pulpotomy between genders is important in all randomized clinical trials [40].

Bleeding time has historically served as a marker for the degree of inflammation [11]. Many practitioners believe that witnessing the degree of pulpal bleeding is an important aspect of success determination rather than relying on preoperative clinical signs and symptoms [36, 41, 42]. In cases of diagnosed irreversible pulpitis, bleeding was managed within 6 min in 84% of instances during partial or full pulpotomy procedures [1, 14]. In our study, bleeding time was categorized into 0–5 min, 6–10 min, and so on till 25 min, and the majority of cases had bleeding time between 6 and 10 min. Also, on comparison with the overall success rate, it was seen that the majority of successful cases had a bleeding time between 6 and 10 min. However, 6 failure cases were also in this category, which points to the synergistic role of other factors like the type of material used, the patient’s physiological response, and clinician skills. In the study by Tan et al., it was concluded that the time needed to achieve hemostasis did not influence the outcome after pulpotomy [43]. Similar results were validated by Linsuwanont in their study on full pulpotomy [44].

Possible reasons for failure in our study could be difficulties and uncertainties in diagnosing pulpal status, especially in younger children, and assessing the extent of inflammation. The quality of coronal restoration is another critical factor for the success of pulpotomy. In all the successful cases, intact restoration without any secondary caries was seen. However, three patients in the TheraCal LC group reported pain in association with the fracture of the restoration, leading to failure of those cases.

The present randomized controlled trial was an exploratory study to compare the efficacy of TheraCal LC and MTA in pulpotomy of permanent molar teeth. However, this study has certain limitations. Attrition of some cases occurred during the follow-up period. Blinding of the operator was not feasible due to the inherent differences in the physical characteristics of the two materials used. The sample size calculation was based solely on the primary outcome, and stratification for potential prognostic factors was not performed, as this would have required a substantially larger sample size. Furthermore, the follow-up duration of 12 months may be insufficient to adequately assess the long-term clinical success of pulpotomy materials. In addition, patient-reported outcomes, such as perception and satisfaction, were not included as parameters in determining treatment success.

The strengths of this study include the use of strict inclusion and exclusion criteria, which helped maintain homogeneity of the study population. Randomization and allocation concealment minimized selection bias, while all treatments were performed under absolutely aseptic conditions to reduce the risk of contamination. In addition, calibration of the operator ensured consistency in the clinical procedures and minimized operator-related variability, thereby strengthening the internal validity of the findings.

To ensure external validity, an adequate sample size was calculated, and subjects were taken from a representative population. For maintaining internal validity, the methods and procedures carried out were standardized, appropriate blinding was carried out, and proper concealment was ensured. Since all patients in this study were treated in an environment conducive to pediatric cooperation, caution should be exercised when extrapolating these findings to other clinical settings. It is noteworthy that the operator’s qualifications had no discernible impact on the treatment outcome; nevertheless, the operator had already received training and calibration, which removed the learning curve that comes with using new material.

## Conclusion

This study concluded that, based on a 12-month clinical and radiographic follow-up, MTA can be considered a better material than TheraCal LC for pulpotomy in permanent molars with symptomatic irreversible pulpitis. However, more multicenter trials in the future with larger sample sizes will be needed to further confirm these findings.

## Clinical trial registration

https://clinicaltrials.gov/ (NCT05733468).
